# Serum obestatin level strongly correlates with lipoprotein subfractions in non-diabetic obese patients

**DOI:** 10.1186/s12944-018-0691-y

**Published:** 2018-03-05

**Authors:** Anita Szentpéteri, Hajnalka Lőrincz, Sándor Somodi, Viktória Evelin Varga, György Paragh, Ildikó Seres, György Paragh, Mariann Harangi

**Affiliations:** 1 Department of Internal Medicine, Faculty of Medicine, University of Debrecen, Nagyerdei krt. 98, Debrecen, H-4032 Hungary; 2 Department of Cell Stress Biology, Department of Dermatology, Roswell Park Cancer Institute, Buffalo, NY USA

**Keywords:** Obestatin, Metabolic syndrome, Diabetes, Obesity, Hyperlipidemia

## Abstract

**Background:**

Obestatin is a ghrelin-associated peptide, derived from preproghrelin. Although many of its effects are unclear, accumulating evidence supports positive actions on both metabolism and cardiovascular function. To date, level of obestatin and its correlations to the lipid subfractions in non-diabetic obese (NDO) patients have not been investigated.

**Methods:**

Fifty NDO patients (BMI: 41.96 ± 8.6 kg/m^2^) and thirty-two normal-weight, age- and gender-matched healthy controls (BMI: 24.16 ± 3.3 kg/m^2^) were enrolled into our study. Obestatin level was measured by ELISA. Low-density lipoprotein (LDL) and high-density lipoprotein (HDL) subfractions, intermediate density lipoprotein (IDL) and very low-density lipoprotein (VLDL) levels and mean LDL size were detected by nongradient polyacrylamide gel electrophoresis (Lipoprint).

**Results:**

Serum level of obestatin was significantly lower in NDO patients compared to controls (3.01 ± 0.5 vs. 3.29 ± 0.6 μg/ml, *p* < 0.05). We found significant negative correlations between the level of obestatin and BMI (*r* = − 0.33; *p* < 0.001), level of serum glucose (*r* = − 0.27, *p* < 0.05), HbA1c (*r* = − 0.38; *p* < 0.001) and insulin (*r* = − 0.34; *p* < 0.05). Significant positive correlation was found between obestatin level and the levels of ApoA1 (*r* = 0.25; *p* < 0.05), large HDL subfraction ratio and level (*r* = 0.23; *p* < 0.05 and *r* = 0.24; *p* < 0.05), IDL (*r* = 0.25 *p* < 0.05) and mean LDL size (*r* = 0.25; *p* < 0.05). Serum VLDL ratio and level negatively correlated with obestatin (*r* = − 0.32; *p* < 0.01 and *r* = − 0.21; *p* = 0.05). In multiple regression analysis obestatin was predicted only by VLDL level.

**Conclusions:**

Based on our data, measurement of obestatin level in obesity may contribute to understand the interplay between gastrointestinal hormone secretion and metabolic alterations in obesity.

## Background

Obesity is one of the leading causes of morbidity and mortality in the world. Globally, the prevalence of obesity has risen at an alarming rate over the past two decades [1]. Numerous studies have shown a clear relationship between obesity and risk of developing cardiovascular disease (CVD). A follow-up analysis from the Framingham study demonstrated high body mass index (BMI) as an independent risk factor for coronary artery disease (CAD), stroke, and overall CVD death [2]. Dyslipidemia is frequently associated to obesity and a well-known risk factor of CVD. The typical dyslipidemia associated with obesity consists of increased triglycerides (TG) and free fatty acid (FFA), decreased high-density lipoprotein-cholesterol (HDL-C) with HDL dysfunction and normal or slightly increased low-density lipoprotein-cholesterol (LDL-C) with increased small dense LDL. The concentration of plasma apolipoprotein (apo) B is also often increased [3, 4].

In the last few decades, it has been recognized that adipose tissue is a highly active metabolic and endocrine organ, and that secreted hormon-like proteins (adipokines) are important for metabolic homeostasis including lipid metabolism [5, 6]. However, the regulatory effect of further proteins secreted by other tissues such as gastrointestinal tract has not been clarified.

Obestatin, a recently identified anorexigenic gut hormone, is a 23 amino acid peptide derived from the C terminal portion of the preproghrelin precursor [7]. There have been many contradicting reports regarding the role of obestatin in humans. Obestatin has opposite action to ghrelin on food intake and plays a role in energy balance [8]. Studies on the obestatin/ghrelin ratio in the gastrointestinal tract and plasma are associated with some diseases such as irritable bowel syndrome [9], obesity [10] and type 2 diabetes mellitus [11]. Plasma obestatin concentrations were negatively correlated with body mass index, insulin resistance index, and plasma leptin concentrations in obesity [12]. Fasting plasma concentration of obestatin, but not of ghrelin, was found to be reduced in insulin resistance and is positively associated with whole body insulin sensitivity in nondiabetic humans [13]. Therefore, obestatin may be a nutritional marker reflecting body adiposity and insulin resistance.

Although a previous study reported that it may also regulate lipid metabolism by inhibiting lipolysis [14], to date, the association of serum obestatin levels with the lipid subfractions has not been studied. Therefore, we aimed to measure the level of serum obestatin and evaluate its correlations to the lipid fractions and subfractions in non-diabetic obese (NDO) patients. We also investigated the possible associations between the concentration of obestatin and the HDL function characterized by HDL-linked anti- and pro-atherogenic enzymes: human paraoxonase-1 (PON1) and myeloperoxidase (MPO).

## Methods

### Study population

We enrolled fifty non-diabetic obese patients that were referred to our obesity outpatient clinic at Department of Internal Medicine, Faculty of Medicine, University of Debrecen, Hungary, and thirty-two healthy volunteers matched in sex and age. All participants provided written informed consent. The study protocol was approved by the Ethical Committee of University of Debrecen and the study was carried out in accordance with the Declaration of Helsinki. Obesity was defined as BMI ≥ 30 kg/m^2^. Participants with active liver or endocrine disease (including any types of diabetes mellitus), cardiovascular disease, renal impairment or malignancy were excluded. Further exclusion criteria were pregnancy, lactation, current smoking, and alcoholism or drug dependence. Neither obese subjects nor lean healthy controls were taking lipid lowering, hyperglycemic, anti-inflammatory, antithrombotic medications or dietary supplements. None of participants were on antihypertensive treatment with exception of ten obese patients, who were on diuretics (indapamide) because of mild hypertension.

### Sample collection and laboratory measurements

All venous blood samples were collected after 12-h of fasting. The routine laboratory parameters including fasting glucose, fructose amine, high sensitive C-reactive protein (hsCRP), total-cholesterol, triglyceride, HDL-C, LDL-C, apoAI, apoB and lipoprotein(a) levels were determined from fresh sera with Cobas c501 analyzer (Roche Ltd., Mannheim, Germany) according to the manufacturer’s instruction. To check non-diabetic status in study participants, we applied a routine 75 g oral glucose tolerance test (OGTT) after an overnight fast. At the same time, hemoglobin A1c (HbA1c) and fasting insulin were also performed according to the standard laboratory techniques. Homeostasis model assessment – insulin resistance (HOMA-IR) was calculated with the formula of Matthews et al. [15]. Sera were kept frozen at − 70 °C for subsequent lipoprotein subfraction analysis and for enzyme-linked immunosorbent assay (ELISA) measurements.

### Lipoprotein subfraction analyses

HDL subfractions were detected by an electrophoretic method on polyacrylamide gel with the Lipoprint System (Quantimetrix Corp., CA, USA) according to the manufacturer’s instructions.Concisely, 25 μl sera were added to the polyacrylamide gel tubes along with 300 μl loading gel solution. The tubes contained Sudan Black as a lipophilic dye and were photopolimerized at room temperature for 30 min. Electrophoresis with tubes containing sera samples or the manufacturer’s quality controls were performed at a constant of 3 mA/tube for 50 min. Each electrophoresis chamber contained a quality control provided by the manufacturer (Liposure Serum Lipoprotein Control, Quantimetrix Corp., CA, USA). Subfraction bands were scanned with an ArtixScan M1 digital scanner (Microtek International Inc., CA, USA) and were identified by their mobility (Rf) using VLDL+LDL as the starting (Rf 0.0) and albumin as the ending (Rf 1.0) reference point.

Ten HDL subfractions were differentiated between VLDL+LDL and albumin peaks, and were grouped into three major classes: large (from HDL1 to HDL3), intermediate (from HDL4 to HDL7) and small (HDL8 to HDL10) HDL subfractions. Cholesterol concentrations of the HDL particle subsets were calculated with Lipoware software (Quantimetrix Corp., CA, USA) by multiplying the total HDL-C concentration of the samples by the relative area under the curve (AUC) of the subfraction bands.

LDL subfractions were also determined using Lipoprint System (Quantimetrix Corp., CA, USA) according to the manufacturer’s instructions. 25 μl of serum samples were added to polyacrylamide gel tubes along with 200 μl a loading gel solution containing Sudan Black as a lipophilic dye. The sample loading gel mixture was photopolymerized for 30 min at room temperature prior to electrophoresis at a constant of 3 mA/tube for 1 h.

Lipoprotein fractions (bands) were identified after electrophoresis by their mobility (Rf) using VLDL as the reference point (Rf 0.0) and HDL as the ending reference point (Rf 1.0). In between, up to seven LDL subfractions were distributed. The percentages of the area under the curve (AUC%) for the VLDL, Midbands (C, B and A; comprising primarily IDL), LDL and HDL peaks, as well as mean LDL size (nm) were calculated with Lipoware computer software (Quantimetrix Corp., CA, USA). Proportion of large LDL (large LDL %) was defined as the sum of the percentage of LDL1 and LDL2, whereas proportion of small LDL (small-dense LDL %) was defined as the sum of LDL3-LDL7. Cholesterol concentrations of LDL subfractions were determined by multiplying the relative AUC of subfractions by total cholesterol concentration of the sample. Calculated total LDL-C is comprised of the sum of the cholesterol in Midbands C through A and LDL subfractions (LDL1-LDL7); and correlates strongly with the directly measured LDL-C [16].

### Determination of human paraoxonase-1 enzyme activities

PON1 paraoxonase activity was analyzed on a microtiter plate by a kinetic, semi-automated method using paraoxon (O,O-diethyl-O-p-nitrophenyl-phosphate, Sigma Aldrich) as a substrate. PON1 arylesterase activity was assayed with a phenylacetate substrate (Sigma Aldrich) and the hydrolysis of phenylacetate was monitored at 270 nm [17].

### ELISA measurements

Plasma human obestatin was determined by EIA kit (Yanaihara Institute Inc., Shizuoka, Japan). Intra- and inter-assay coefficients of variations (CV) were 3.5–9.9% and 5.6–9.0%, respectively. MPO and oxidized LDL (oxLDL) concentrations were determined by a commercially available ELISA kits (R&D Systems, Minneapolis, MN, USA and Mercodia AB, Sweden, respectively) with 6.6–7.7 CV% intra-, and 6.5–9.4 CV% inter-assay (MPO) and 5.5–7.3 CV% intra-, and 4–6.2 CV% inter-assay precision (oxidized LDL). All assays were performed according to the recommendation of the manufacturer.

### Statistical methods

Statistical analysis was performed by STATISTICA version 8.0 (Statsoft Inc., Tulsa, OK, USA). The normality of data distribution was tested by Kolmogorov-Smirnov test. Data were presented by descriptive analysis (means±SD in case of normal distribution, or medians [lower quartile – upper quartile] in the case of non-normal distribution). Comparisons between groups were performed by Student’s unpaired t-test in case of normally distributed variables and by Mann-Whitney U-test in case of variables with non-normal distribution. Correlations between continuous variables were assessed by linear regression analysis using Pearson’s test. Since the distribution of some variables of interest became normal upon base-10 logarithm transformation, we used the log values for correlation analyses. Multiple regression analysis was performed to determine which variables best predicted obestatin concentrations. Results were considered to be significant at the level of *p* < 0.05.

## Results

Anthropometric data and laboratory characteristics of study participants are summarized in Table 1 The NDO patients had extremely high BMI and slightly elevated hsCRP level compared to lean individuals. Although, there were several other differences in the laboratory parameters in NDO patients compared to lean controls, these data were found to be in the physiological range. Plasma triglyceride and lipoprotein(a) concentrations were found significantly higher, while the levels of HDL-C and apoAI were significantly lower in the obese group compared to normal weight controls. HbA1C level was significantly higher in the obese individuals compared to the controls. Fasting glucose was in normal range in both groups and the blood glucose levels at 120 min of OGTT were not elevated in the obese group. On the basis of these laboratory parameters the obese patients involved into this study have neither diabetes nor impaired glucose tolerance.

**Table 1:** Anthropometric and routine laboratory parameters of study participants

	Obese (*n* = 50)	Control (*n* = 32)	*P*
Gender (F/M)	43 / 7	27 / 5	ns
Age (yrs)	44.20 ± 13.50	41.78 ± 5.97	ns
Body mass index (kg/m^2^)	41.96 ± 8.63	24.47 ± 2.51	< 0.001
Waist circumference (cm)	119.76 ± 16.87	83.62 ± 9.25	< 0.01
hsCRP (mg/l)	8.24 (3.2–13.09)	1.57 (0.6–2.94)	< 0.001
Fructose amine (μmol/l)	225.32 ± 27.95	229.0 ± 11.65	ns
Thyroid stimulating hormone (mU/l)	1.98 ± 0.98	1.93 ± 1.15	ns
Lipid parameters			
Triglyceride (mmol/l)	1.4 (1.1–2.0)	1.0 (0.75–1.39)	< 0.01
Total cholesterol (mmol/l)	5.04 ± 0.83	5.07 ± 0.78	ns
HDL-cholesterol (mmol/l)	1.36 ± 0.33	1.56 ± 0.46	< 0.05
LDL-cholesterol (mmol/l)	3.17 ± 0.74	2.93 ± 0.52	ns
Apolipoprotein A-I (g/l)	1.48 ± 0.24	1.68 ± 0.31	< 0.01
Apolipoprotein B (g/l)	0.86 ± 0.20	0.94 ± 0.18	ns
Lipoprotein (a) (mg/l)	248 (120–586)	70 (30–214)	< 0.001
Carbohydrate parameters			
Hemoglobin A1c (%)	5.76 ± 0.54	5.07 ± 0.33	< 0.001
Fasting glucose (mmol/l)	4.90 ± 0.75	4.82 ± 0.48	ns
OGTT 120 min	7.00 ± 2.01		
Fasting insulin (mU/l)	21.01 ± 15.91		
HOMA-IR	3.75 (2.4–6.52)		
Inflammatory and oxidative markers			
Obestatin (μg/ml)	3.01 ± 0.5	3.29 ± 0.6	< 0.05
Oxidized LDL (U/L)	46.8 ± 9.95	41.1 ± 9.57	< 0.01
Paraoxonase activity (U/L)	64.72 (43.79–149.52)	83.03 (47.9–167.4)	ns
Arylesterase activity (U/L)	121.61 ± 23.65	131.1 ± 28.75	ns
Myeloperoxidase (ng/ml)	280 (148.3–386.3)	207.9 (125.8–265.2)	< 0.05

Values are presented as mean ± standard deviation or median (lower quartile - upper quartile). *Abbreviations*: *HDL* high-density lipoprotein, *hsCRP* high sensitive C-reactive protein, *LDL* low-density-lipoprotein, *OGTT* oral glucose tolerant test, *HOMA-IR* homeostasis model assessment insulin resistance, *ns* non-significant

Significantly higher VLDL, large LDL, small LDL and small HDL levels, while significantly lower IDL, mean LDL size, large HDL and intermediate HDL levels were found in NDO patients compared to the control population (Table 2).

**Table 2:** Concentration and ratio of lipoprotein subfractions in non-diabetic obese and lean participants

	Obese (*n* = 50)	Control (*n* = 32)	*P*
VLDL subfraction (mmol/l)	1.165 ± 0.17	0.868 ± 0.17	< 0.001
Midband (IDL) (mmol/l)	1.207 ± 0.31	1.505 ± 0.38	< 0.001
VLDL subfraction (%)	23.3 ± 2.5	17.1 ± 2.3	< 0.001
Midband (IDL) (%)	23.7 ± 3.6	29.6 ± 5	< 0.001
LDL subfractions			
Large LDL (mmol/l)	1.267 (1.06–1.603)	1.047 (0.827–1.344)	< 0.01
Small-dense LDL (mmol/l)	0.091 (0.026–0.155)	0.026 (0–0.052)	< 0.001
Mean LDL size (nm)	26.98 ± 0.31	27.26 ± 0.37	< 0.001
Large LDL (%)	25.8 ± 4.1	21.1 ± 5.8	< 0.001
Small-dense LDL (%)	1.96 ± 1.57	1.05 ± 2.26	< 0.05
HDL subfractions			
Large HDL (mmol/l)	0.284 (0.207–0.388)	0.453 (0.31–0.608)	< 0.001
Intermediate HDL (mmol/l)	0.6594 (0.595–0.828)	0.749 (0.659–0.853)	< 0.05
Small HDL (mmol/l)	0.336 (0.284–0.388)	0.284 (0.246–0.336)	< 0.01
Large HDL (%)	22.5 ± 5.7	29.8 ± 9.0	< 0.001
Intermediate HDL (%)	52.3 ± 3.4	50.8 ± 4.7	ns
Small HDL (%)	25.2 ± 5.9	19.3 ± 5.3	< 0.001

Values are presented as mean ± standard deviation or median (lower-upper quartiles)

*Abbreviations*: *HDL* high-density lipoprotein, *hsCRP* high sensitive C-reactive protein, *IDL* intermediate density lipoprotein, *LDL* low-density-lipoprotein, *OGTT* oral glucose tolerant test, *HOMA-IR* homeostasis, *VLDL* very low-density lipoprotein

Serum level of obestatin was significantly lower in NDO patients compared to controls (3.01 ± 0.5 vs. 3.29 ± 0.6 μg/ml, *p* < 0.05) (Table 1). We found significant negative correlations between obestatin levels and BMI (*r* = − 0.33; *p* < 0.001), serum glucose levels (*r* = − 0.27, *p* < 0.05), HbA1c (*r* = − 0.38; *p* < 0.001) and insulin (*r* = − 0.34; *p* < 0.05; data not shown).

**Fig. 1:**
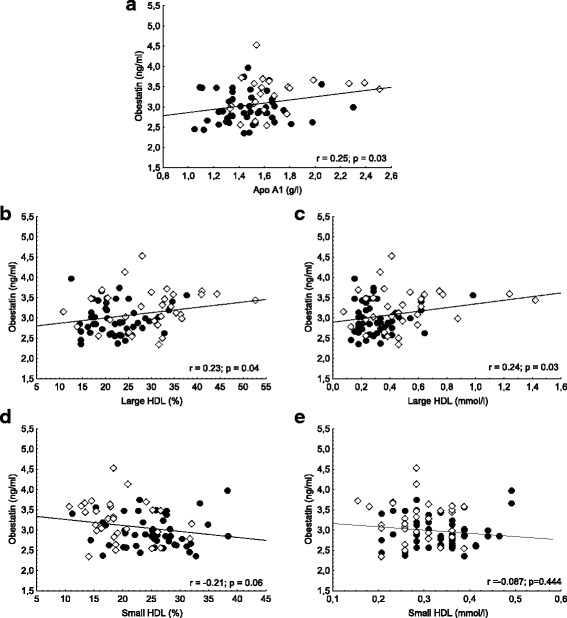
Correlations between serum obestatin level and (**a**) apolipoprotein A1 (ApoA1); **b** large high-density lipoprotein subfraction ratio (large HDL %); **c** large HDL subfraction level (large HDL); **d** small HDL subfraction ratio (small HDL %); and (**e**) small HDL subfraction level (small HDL) in non-diabetic obese (●) and normal weight controls (**◊**)

**Fig. 2:**
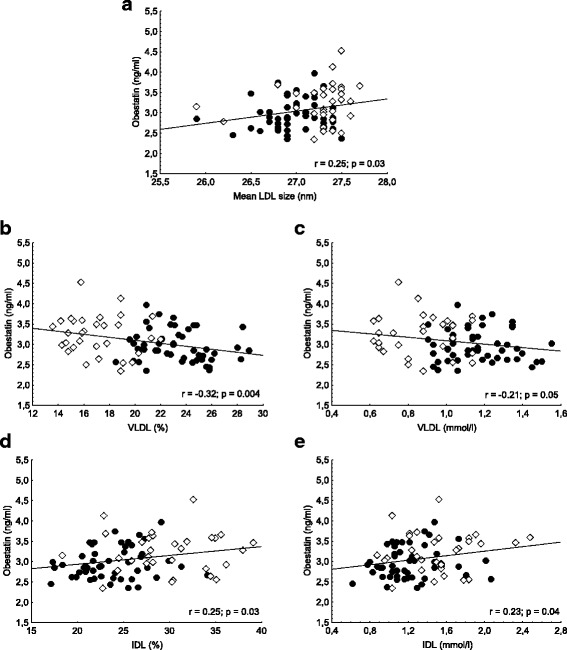
Correlations between serum obestatin level and (**a**) mean low-density lipoprotein size (mean LDL size); **b** very low-density lipoprotein ratio (VLDL %); **c** VLDL level (VLDL); **d** intermediate-density lipoprotein subfraction level (IDL%) and (**e**) IDL level (IDL) in non-diabetic obese (●) and normal weight controls (**◊**)

Significant positive correlation was found between obestatin level and the levels of ApoA1 (*r* = 0.25; *p* < 0.05), the ratio in % of large HDL subfractions (*r* = 0.23; *p* < 0.05) and the level of large HDL subfractions (0.24; *p* < 0.05). Small HDL subfraction ratio in % showed negative, but non-significant correlation with obestatin level (− 0.21; *p* = 0.06), while small HDL level did not show any correlation with obestatin **(**Fig. 1**.**). We detected significant positive correlation between obestatin level and mean LDL size (*r* = 0.25; *p* < 0.05). Significant negative correlations were found between obestatin and ratio of VLDL in % (*r* = − 0.32; *p* < 0.01) and VLDL level (*r* = − 0.21; *p* = 0.05), while there were significant positive correlations between obestatin and ratio of IDL in % (*r* = 0.25; *p* < 0.05) and IDL level (*r* = 0.23; *p* < 0.05) (Fig. 2).

Increased oxLDL and MPO levels were found in NDO patients compared to the control population. PON1 paraoxonase and arylesterase acivities did not differ significantly between patients and controls (Table 1). We could not find significant correlations between obestatin and the levels of MPO and PON1 paraoxonase and arylesterase activities.

In multiple regression analysis obestatin was predicted only by VLDL level (Table 3).

**Table 3:** Multivariate analysis for obestatin as a dependent variable on all study participants

Variable	Beta	*p*-value
Body mass index (kg/m^2^)	0.073	0.09
Glucose (mmol/l)	−0.22	0.80
Hemoglobin A1c (%)	−0.05	0.22
Apolipoprotein A1 (g/l)	−0.28	0.054
VLDL (mmol/l)	−0.29	< 0.05
IDL (mmol/l)	−0.03	0.36
large HDL (mmol/l)	0.413	0.06
small HDL (mmol/l)	0.069	0.69
Mean LDL size (nm)	−0.07	0.73

*Abbreviations*: *HDL* high-density lipoprotein, *IDL* intermediate density lipoprotein, *LDL* low-density-lipoprotein, *OGTT* oral glucose tolerant test, *HOMA-IR* homeostasis

## Discussion

Obestatin acts as an anorectic hormone that decreases food intake, slows gastrointestinal motility and therefore reduces weight gain [12]. Previous studies in humans showed significantly lower plasma obestatin levels in diabetic or non-diabetic obese subjects compared to lean controls but failed to assess diabetes mellitus or impaired glucose tolerance status [18]. We found similar results in our obese subjects without diabetes.

The exact role of obestatin in regulation of lipoprotein levels is not completely clarified.

Some previous studies showed that it may regulate lipid metabolism by inhibiting lipolysis in 3 T3 and human subcutaneous and omental adipocytes isolated from lean and obese individuals and mice on high-fat diet [19, 20]. Obestatin increases AMP kinase phosphorylation leading to enhanced lipolysis in adipocytes [20]. Moreover, administration of N-terminally PEGylated obestatin significantly reduced plasma triglyceride levels in rat [21]. Interestingly obestatin infusion reduced the key lipid transporter ATP-binding cassette A1 expression in cow white adipose tissue [22].

Correlations between obestatin levels and lipoprotein subfraction parameters have to the best of our knowledge have not previously been investigated. We found a significant positive correlation between obestatin level and the levels of ApoA1 and large HDL subfractions, which may indicate a possible connection between the abnormal gastrointestinal response and decreased hepatic ApoA1 expression in obesity. Furthermore, serum VLDL ratio and level negatively correlated with obestatin, which may be explained by the previously described association between the serum level of obestatin and carbohydrate metabolism, since insulin resistance and the higher level of serum glucose result in increased hepatic free fatty acid production leading to elevated VLDL level [23]. Moreover, in multiple regression analysis VLDL level was the only independent predictor of obestatin level. The negative VLDL correlation likely also explains the large HDL subfraction positive correlation to obestatin levels, which was approaching significance (*p* = 0.06) on multiple regression analysis. Increased transport of triglyceride from VLDL to HDL and cholesterol-esther from HDL to VLDL by cholesterol-esther transfer protein lead to the formation of smaller and denser HDL particles with enhanced degradation and lower half lifespan, which results in low total HDL-C levels and a shift towards smaller HDL subfractions [24].

We also investigated the activity of human paraoxonase-1, an antioxidant enzyme mainly associated with smaller HDL particles containing apolipoprotein J (clusterin) [25, 26]. Although both paraoxonase and arylesterase activities of the enzyme tended to be lower in obese subjects, there were no significant differences in enzyme activities between the two study groups, despite the shift towards the smaller HDL subfractions. Furthermore, we found no significant correlation between obestatin levels and PON1 enzyme activity.

The level of another HDL associated, pro-atherogenic enzyme: myeloperoxidase was also investigated. In line with some previous studies [27, 28] we found significantly higher myeloperoxidase level in obese subjects compared to lean controls. Previous data shows that MPO, PON1, and HDL may bind to each other, forming a ternary complex, wherein PON1 partially inhibits MPO activity and MPO inactivates PON1 influencing endogenous oxidative stress and lipid peroxidation during inflammation [29]. In our previous study PON1 arylesterase activity was found to be an independent predictor of MPO levels in overweight hyperlipidemic, lipid-lowering therapy-naive patients [30]. In the nondiabetic obese group there were no significant correlations either between paraoxonase activity and myeloperoxidase level or between obestatin and myeloperoxidase level.

A previous study showed obestatin increased oxLDL binding to macrophages [31]. Although, oxLDL level was significantly higher in obese patients, we could not find significant correlation between the levels of oxLDL and obestatin.

Some limitations of the study can be noted. The power of the study may be reduced because of the relatively small number of obese subjects. Obestatin secretion was found to be pulsatile and displayed an ultradian rhythmicity in a previous study [8]. We investigated fasting serum obestatin levels; however, postprandial levels of obestatin may show altered correlations with quantitative and qualitative parameters of lipoproteins.

## Conclusion

We concluded that decreased level of obestatin may contribute to the development of metabolic syndrome and altered lipoprotein metabolism in obese patients even without disturbed insulin sensitivity. However, obestatin level does not correlate to HDL function markers including PON1 and MPO and has no effect on the level of oxidized LDL. Based on our data, measurement of obestatin level in obesity may contribute to understand the interplay between gastrointestinal hormone secretion and metabolic alterations in obesity.

## Abbreviations

BMI: Body mass index
CAD: Coronary artery disease
CVD: Cardiovascular disease
ELISA: Enzyme-linked immunosorbent assay
FFA: Free fatty acid
HbA1c: Hemoglobin A1c
HDL: High-density lipoprotein
HDL-C: High-density lipoprotein-cholesterol
HOMA-IR: Homeostasis model assessment – insulin resistance
hsCRP: High sensitive C-reactive protein
IDL: Intermediate density lipoprotein
LDL: Low-density lipoprotein
LDL-C: low-density lipoprotein-cholesterol
MPO: Myeloperoxidase
NDO: Non-diabetic obese
OGTT: Oral glucose tolerance test
oxLDL: Oxidized LDL
PON1: Human paraoxonase-1
TG: Triglycerides
VLDL: Very low-density lipoprotein
